# Data on trace element concentrations in coal and host rock and leaching product in different pH values and open/closed environments

**DOI:** 10.1016/j.dib.2019.104053

**Published:** 2019-05-23

**Authors:** Yao Shan, Wenfeng Wang, Yong Qin

**Affiliations:** a School of Safety Engineering, North China Institute of Science and Technology, Yanjiao, 101601, China; b School of Resource and Earth Science, China University of Mining and Technology, Xuzhou, 221116, China

**Keywords:** Coal, Coal host rock, Trace elements, Leaching mechanism

## Abstract

The data presented in this article are related to the research article entitled “Multivariate analysis of trace elements leaching from coal and host rock” [1]. During coal mining, coal and host rock undergo water-rock interaction, leading to release of trace elements to surface and ground water. The batch experiments were designed and implemented to investigate the leaching behavior and mechanisms during the process of water-rock interaction. In different experimental sets, various types of leaching water, open/closed environments, temperature, and initial pH values were used to evaluate their impact on leaching of trace elements. These data could be used to analyze leaching mechanisms of trace elements from coal and host rock, and understand, predict, control trace elements’ contamination to surrounding waters.

**Table:** Specifications table

Subject area	*Geochemistry*
More specific subject area	*Environmental Geochemistry*
Type of data	*Tables*
How data was acquired	ICP-AES (JY38S ICP-AES); ICP-MS (X-Series ICP-MS- Thermo Electron Co.)
Data format	*(Raw data: important environmental concern elements were tested)*
Experimental factors	*Solid samples for testing (coal and host rock) were digested.*
Experimental features	*Host rock and coal samples were leaching using a batch mode, samples were collected using syringe. Different pH values and open/closed environments were applied in the experiments.*
Data source location	*Samples of coal, host rock for testing and leaching was collected in Xuzhou-Datun coal mine district, Jiangsu province, China.*
Data accessibility	*The data are available only in this article.*
Related research article	*Y. Shan, W. Wang, Y. Qin, L. Gao. Multivariate analysis of trace elements leaching from coal and host rock, Groundwater for Sustainable Development., 8,**2019**, 402–412*[1]*.*

**Table:** 

**Value of the data** • The data show major and trace element concentrations in a Chinese coal mine district, which can be used to compared with other coal mine districts. • The data show leaching behavior of trace elements from coal and host rock, which can be used to investigate the environmental impact of coal mine water. • The data show the impact of pH and open/closed conditions on leaching behavior, which can be used to analyze the leaching mechanisms of trace elements from coal and host rock. • The data could be used to understand, predict, and control the contamination of trace elements on surrounding waters.

## 1 Data

Table 1, Table 2 show concentrations of major and trace elements in coal samples, and Table 3, Table 4 show concentrations in host rock samples after the first and second sampling and testing. Analysis of ICP-AES, ICP-MS were applied in the first the second testing, respectively. 26 and 16 elements were analyzed in the first and second testing, respectively.

**Table 1:** Major and trace element concentrations in coal samples (mg/kg) (First sampling and test).

No.	Sample type	From coal seam	Mo	Zn	Pb	Co	Ni	Ba	Mn	Cr	Ga	V	Nb	Be	Cu
1	Coal	No.2	2.1	13.42	29.65	6.11	13.71	125.5	68.54	26.11	12.42	95.27	13.61	4.64	55.24
2	Coal	No.2	2.1	23.5	38.54	7.5	14.74	182.9	66.26	30.27	14.9	95.98	13.96	2.05	76.72
3	Coal near roof	No.7	1.78	17.75	57.51	6.29	9.23	129.2	24.27	56.54	13.14	117.7	23.03	6.74	74.9
4	Coal near roof	No.7	0.84	412.1	27.87	8.5	21.35	42	31.86	30.35	10.58	126.2	16.75	5.83	50.68
5	Coal with pyrite nodule	No.7	1.06	20.69	31.42	5.42	9.79	57.9	58.11	12.85	10.89	18.9	9.68	1.06	49.55
6	Coal with pyrite nodule	No.7	2.06	54.54	41.5	8.92	27.33	242.7	35.11	42.01	20.45	198.4	28.84	3.87	65.83
7	Coal	No.7	1.26	17.1	18.97	4.25	7.28	175	107.6	19.36	6.78	29.36	7.21	1.06	34.97
8	Coal	No.7	1.09	27.56	29.05	5.23	11.24	223.3	132.8	20.99	12.26	41.53	9	1.49	57.61
9	Coal	No.7	1.49	17.98	30.11	5.08	10.22	192.1	69.52	26.09	14.03	73.14	11.27	1.74	62.92
10	Coal	No.7	0.54	17.42	13.25	1.81	5.47	74.2	32.3	9.38	9	6.56	2.58	0.44	24.34
11	Coal near floor	No.7	1.14	21.49	32.02	7.89	13.29	178.4	108	48.18	13.9	58.53	12.69	3.19	54.86
12	Coal near floor	No.7	1.17	9.16	17.79	4.39	12.96	127	78.41	9.27	12.51	15.56	4.14	1.38	33.31
13	Coal near roof	No.9	0.75	8.91	13.04	1.92	3.79	128.4	59.51	11.57	6.54	27.76	5.15	0.55	45.83
14	Coal near roof	No.9	1.35	18.5	29.65	6.15	10.59	114.7	29.85	27.79	12.8	67.23	15.09	2.19	70.55
15	Coal near roof	No.9	0.88	10.3	18.38	3.03	4.68	63.1	53.99	15.11	8.41	22.09	6.93	0.91	44.56
16	Coal	No.9	1.49	25.24	25.5	4.87	10.82	237.3	102.6	21.77	11.23	44.81	8.93	1.1	69.28
17	Coal	No.9	1.39	31.26	34.39	6.64	14.86	184.6	99.7	33.86	14.69	50.41	9.92	1.34	68.07
	Sample type	From coal seam	Ti	Sc	Sr	Fe%	Mg%	Ca%	Al%	Na%	K%	As	Hg	Se	Cd
1	Coal	No.2	1999	9.21	102.6	0.54	0.14	1.66	3.33	0.1	0.16	5.58	0.07	0.12	0.03
2	Coal	No.2	2263	8.68	101.8	0.9	0.17	0.76	5.03	0.14	0.3	7.69	0.1	0.13	0.05
3	Coal near roof	No.7	3498	21.02	86.4	3.38	0.1	0.18	5.83	0.04	0.12	1.27	0.07	0.18	0.08
4	Coal near roof	No.7	897	10.72	41.1	1.89	0.05	0.15	2.8	0.03	0.06	5.2	0.07	0.09	0.2
5	Coal with pyrite nodule	No.7	1307	4.62	59.5	3	0.04	0.18	3.27	0.04	0.03	4.63	0.06	0.13	0.12
6	Coal with pyrite nodule	No.7	1867	10.28	120.4	3.29	0.2	0.19	4.23	0.11	0.65	7.26	0.13	0.16	0.08
7	Coal	No.7	1184	4.45	141.9	0.64	0.24	1.1	2.69	0.06	0.16	3.87	0.04	0.07	0.02
8	Coal	No.7	1565	5.46	150.3	0.79	0.36	1.28	2.84	0.08	0.52	4.16	0.07	0.11	0.08
9	Coal	No.7	1763	6.79	145.5	0.7	0.15	0.79	3.22	0.1	0.25	5.02	0.07	0.11	0.22
10	Coal	No.7	266	1.87	108.6	0.15	0.05	0.6	0.57	0.03	0.03	1.74	0.03	0.05	0.02
11	Coal near floor	No.7	2087	13.09	98	0.79	0.21	1.24	3.92	0.05	0.45	6.12	0.07	0.12	0.05
12	Coal near floor	No.7	420	2.46	132	0.5	0.09	1.79	1.11	0.04	0.03	2.15	0.04	0.08	0.1
13	Coal near roof	No.9	681	2.83	90.5	0.46	0.1	0.77	0.89	0.04	0.06	2.25	0.04	0.06	0.02
14	Coal near roof	No.9	2044	8.39	94	0.81	0.1	0.15	3.14	0.05	0.16	5.6	0.06	0.11	0.04
15	Coal near roof	No.9	1269	3.66	89.1	0.53	0.13	0.33	1.61	0.04	0.06	2.3	0.03	0.07	0.02
16	Coal	No.9	1299	5.23	183.9	0.96	0.21	1.69	2.92	0.11	0.22	4.19	0.07	0.1	0.04
17	Coal	No.9	1845	7.57	123.2	0.96	0.23	1.17	3.21	0.08	0.3	3.79	0.06	0.1	0.05

**Table 2:** Major and trace element concentrations in coal samples (mg/kg) (Second sampling and test).

No.	Sample type	From coal seam	Be	Sc	Ti	V	Cr	Mn	Co	Ni
1	Coal	No.2	2.54	16.6	4292	135	81.2	44.66	14.5	36.6
2	Coal	No.2	1.18	1.88	658	1.76	20.4	46.9	3.82	9.31
3	Coal near roof	No.7	2.22	16.9	4192	119	66.9	32.54	8.21	27.1
4	Coal near roof	No.7	2.67	17.2	4266	181	101	44.78	15.9	34.9
5	Coal	No.7	2.67	16.7	7430	133	110	27.9	5.1	29.1
6	Coal	No.7	3.29	16.7	7210	145	98.5	27.35	5.61	30.5
7	Coal	No.7	2.47	1.38	344	12.5	6.78	29.8	11.8	11.5
8	Coal	No.7	0.6	2.37	922	9.95	8.62	47.1	6.84	57
9	Coal	No.7	0.62	2.47	975	4.91	25.2	59	6.77	14.2
10	Coal	No.7	1.23	1.86	650	8.53	40.4	42.5	3.82	10.1
11	Coal	No.7	0.64	2.43	950	16.9	13.4	29.3	6.89	14.1
12	Coal near floor	No.7	2.91	18	4431	149	84.7	44.43	15.7	33.5
13	Coal near floor	No.7	0.67	2.64	1037	1.72	8.37	47.1	7.65	17.5
14	Coal near roof	No.9	0.56	2.44	924	8.95	10.4	46.4	6.93	13.5
15	Coal	No.9	2.21	17.1	4336	135	135	31.69	8.05	26.7
16	Coal	No.9	2.66	17.1	4301	210	107	45.31	15.3	33.9
17	Coal	No.9	0.59	2.56	949	10.5	15.7	49.6	6.97	14.2
18	Coal	No.9	0.58	2.39	941	6.48	24.6	52	6.98	14.4
19	Coal near floor	No.9	2.96	18.3	4577	124	88.7	46.45	15.8	36.5
20	Coal near floor	No.9	0.6	2.44	931	7.37	16.1	44.7	7.04	26.4
	Sample type	From coal seam	Cu	Zn	Ga	Sr	Nb	Mo	Ba	Pb
1	Coal	No.2	66.1	64.5	21.3	108	13.5	0.32	248	26.42
2	Coal	No.2	10.1	13.3	4.73	48.4	4.35	0.59	47.48	9.75
3	Coal near roof	No.7	54.6	55.3	21.1	72	13	0.5	245	22.26
4	Coal near roof	No.7	58.9	57.5	21.1	108	13.4	0.43	238	26.19
5	Coal	No.7	33	30.4	23.8	60.2	20.8	0.42	244	23.53
6	Coal	No.7	38.5	39	23.6	95.6	20.3	0.33	229	24.88
7	Coal	No.7	7.69	12.2	5.06	48	1.34	0.79	66.9	6.07
8	Coal	No.7	12.1	19.2	14.6	708	3.35	0.79	404.6	4.73
9	Coal	No.7	11.8	1.92	15	690	3.37	0.82	414.8	4.49
10	Coal	No.7	9.91	6.2	4.86	56	4.34	0.71	50.2	10
11	Coal	No.7	11.7	1.64	15.1	704	3.46	0.81	408.2	4.86
12	Coal near floor	No.7	51.4	56.3	21.6	116	13.5	0.35	236	27.67
13	Coal near floor	No.7	13.5	2.35	16.3	731	3.73	0.83	442.9	5.33
14	Coal near roof	No.9	11	1.8	14.8	707	3.42	0.74	409.4	4.16
15	Coal	No.9	53.6	55.3	21.2	70.3	13.6	0.72	239	22.99
16	Coal	No.9	61	68.4	22	93.1	13.3	0.39	252	27.42
17	Coal	No.9	11.4	3.98	15.5	734	3.52	0.83	433.3	4.12
18	Coal	No.9	11.8	9.33	14.9	697	3.37	0.88	412.8	5.55
19	Coal near floor	No.9	64.2	72.5	23.5	112	14.1	0.46	275	29.68
20	Coal near floor	No.9	10.9	6.92	14.5	678	3.43	0.75	397.6	4.72

**Table 3:** Major and trace element concentrations in coal host rock samples (mg/kg) (First sampling and test).

No.	Sample type	From coal seam	Mo	Zn	Pb	Co	Ni	Ba	Mn	Cr	Ga	V	Nb	Be	Cu
1	Roof	No.2	3.28	87.85	60.43	23.83	38.64	490.7	1659	61.46	24.46	90.73	18.23	2.4	57.45
2	Floor	No.2	2.41	31.71	57.51	9.54	14.9	244.9	25.57	55.65	20.99	95.04	19.91	2.81	63.22
3	Main Roof	No.7	2.62	50.67	64.03	13.73	25.23	203.8	239.8	57.89	21.71	114	24.2	3.73	76.24
4	Roof	No.7	3.79	34.92	77.08	12.4	11.81	223.6	40.68	74.36	30	169.4	45.33	2.96	73.57
5	Roof	No.7	3.33	23.24	71.15	7.27	19.36	135.1	25.18	82.6	25.16	127.4	25.26	3.09	82.22
6	Roof	No.7	1.7	54.27	54.54	9.68	22.58	250.4	194.3	52.94	19.04	130.7	22.71	3.65	73.45
7	Roof	No.7	2.03	81.65	60.48	13.68	29.18	483.3	142.4	64.56	22.06	79.4	16.03	2.1	71.15
8	Floor	No.7	3.65	88.44	68.18	7.86	11.91	424.3	35.47	93.35	21.06	98.31	22.76	2.1	49.53
9	Floor	No.7	2.43	21.42	62.25	6.32	14.7	170.5	20.89	46.09	24.68	98.69	22.15	1.91	64.98
10	Floor	No.7	2.68	28.07	75.89	5.93	15.66	424.1	50	76.35	23.09	97.4	21.12	2.37	69.52
11	Roof	No.9	2.73	148.7	60.48	10.87	29.78	505.8	80.51	64.55	21.33	195.8	24.13	2.58	78.72
	Sample type	From coal seam	Ti	Sc	Sr	Fe%	Mg%	Ca%	Al%	Na%	K%	As	Hg	Se	Cd
1	Roof	No.2	4463	15.61	139	4.5	0.81	0.43	10.15	0.71	1.38	19.05	0.22	0.28	0.13
2	Floor	No.2	5315	16.91	76.7	1.1	0.18	0.23	11.6	0.24	0.31	14.88	0.15	0.2	0.09
3	Main Roof	No.7	4471	16.48	189.7	2.27	0.48	0.58	8.88	0.17	0.95	6.78	0.08	0.15	0.06
4	Roof	No.7	9395	13.32	134.4	1.23	0.19	0.14	12.22	0.09	0.45	13.6	0.13	0.17	0.06
5	Roof	No.7	4092	18.86	103.2	0.93	0.33	0.24	13.5	0.08	0.73	14.69	0.15	0.18	0.04
6	Roof	No.7	3368	14.87	189.7	3.26	0.57	0.91	6.37	0.1	0.59	4.8	0.09	0.15	0.06
7	Roof	No.7	3526	11.92	190.2	5.89	0.8	0.16	8.1	0.29	1.48	13.93	0.16	0.26	0.13
8	Floor	No.7	4559	19.37	124.7	1.73	0.4	0.17	13.76	0.09	1.42	15.32	0.16	0.19	0.09
9	Floor	No.7	3844	13.22	143.8	1.23	0.26	0.16	11.32	0.11	0.57	8.37	0.11	0.15	0.04
10	Floor	No.7	4867	16.55	187.4	0.82	0.37	0.12	11.19	0.17	1.25	7.75	0.09	0.12	0.04
11	Roof	No.9	2928	12.58	180	2.04	0.4	0.12	8.63	0.15	1.45	14.73	0.17	0.22	0.08

**Table 4:** Major and trace element concentrations in coal samples (mg/kg) (Second sampling and test).

No.	Sample type	From coal seam	Be	Sc	Ti	V	Cr	Mn	Co	Ni
1	Roof	No.2	2.84	15.2	6368	153	63	26.71	4.86	24.6
2	Floor	No.2	2.75	16.8	4224	119	137	44.28	14.5	34.6
3	Main Roof	No.7	0.61	2.26	918	22.7	9.45	43.4	6.66	14.8
4	Roof	No.7	0.58	2.37	931	11	12.3	28.2	6.75	13.9
5	Roof	No.7	4.01	19.3	4288	181	71.5	26.08	30.6	24.3
6	Roof	No.7	0.58	2.37	936	21.4	19.6	38.8	6.74	13.7
7	Roof	No.7	2.87	21.5	6989	138	96.3	16.36	6.18	26.3
8	Roof	No.7	3.01	19.3	4750	176	108	53.29	16.2	38
9	False Roof	No.7	0.57	2.45	947	15.1	14.4	42.5	7.08	14.4
10	Floor	No.7	1.99	13.7	4561	172	84.3	45.26	8.24	16.3
11	Floor	No.7	0.59	2.44	933	16.7	13	40.6	6.77	13.6
12	Floor	No.7	0.6	2.46	933	19.7	10.9	42.9	6.88	13.6
13	Floor	No.7	2.54	17.2	4233	101	89.5	49.83	14.2	35.4
14	Roof	No.9	2.83	17.7	4319	116	104	46.35	15.2	37.4
15	Roof	No.9	0.65	2.44	971	4.43	18.7	23.1	6.99	13.3
16	Floor	No.9	2.81	14.9	6462	119	75.1	26.4	5.25	77.9
	Sample type	From coal seam	Cu	Zn	Ga	Sr	Nb	Mo	Ba	Pb
1	Roof	No.2	34.7	102	21	74.4	18.7	0.25	228	29.4
2	Floor	No.2	59.4	63.8	21.4	95.4	13.2	0.55	256	27.66
3	Main Roof	No.7	10.5	9.75	14.6	703	3.36	0.78	399.8	4.47
4	Roof	No.7	11	3.08	14.6	688	3.38	0.81	398.8	5.23
5	Roof	No.7	126	49.9	16.8	123	17.7	1.82	116	64.56
6	Roof	No.7	11	2.33	14.4	670	3.38	0.78	391.4	4.24
7	Roof	No.7	65.8	43.7	20	98.1	28.1	1.51	101	42.51
8	Roof	No.7	66.9	68.4	23.8	116	14.6	0.38	265	29.08
9	False Roof	No.7	11.6	4.54	14.8	700	3.5	0.74	407.5	4.48
10	Floor	No.7	38.2	148	22.6	95.8	13.8	2.69	335	31.18
11	Floor	No.7	10.6	2.8	14.6	707	3.41	0.82	404.5	5.26
12	Floor	No.7	11.5	2.61	16.8	697	3.43	0.83	472.9	5.13
13	Floor	No.7	62.3	64.2	21.2	94.3	13.3	0.43	250	27.9
14	Roof	No.9	74.7	108	21.8	114	13.5	0.58	252	27.72
15	Roof	No.9	11.6	3.13	14.9	688	3.56	0.83	402.7	4.78
16	Floor	No.9	34.3	27.9	20.8	83.4	18.9	0.32	216	21.77

Selected coal and host rock samples were used in the leaching experiments, to simulate leaching behavior of the trace elements. Table 5, Table 6 show leaching behavior of trace elements from coal and host rock, respectively, which were tested by ICP-MS. In the coal and host rock leaching experiments, different conditions, including type of leaching water, initial pH values, open/closed environments, and temperature, were set.

**Table 5:** Trace element concentrations in leachate of coal samples (μg/L).

Experimental set	Sampling time(h)	pH	Al	Si	P	Be	B	Ag	Sc	Ti	V	Cr	Mn	Fe	Co
1	2	–	103.9	3604	49.2	0	211.7	36290	2	2.63	2.76	1.06	1.77	38.1	0.94
	6	8.45	127.5	3658	36.9	0	217.5	36150	1.88	2.73	2.94	0.97	1.48	35.5	1.08
	24	8.67	150.9	3773	22.4	/	223	35680	1.91	2.29	2.9	0.97	0.65	35.6	1.07
	48	8.78	138.4	4140	23.7	0.005	227.8	35450	2.29	3.77	3.09	1.38	0.51	33.5	1.93
2	2	–	326.1	864.5	15.9	0.002	41.48	517.8	0.44	1.89	0.99	0.92	4.28	64	0.55
	6	8.78	225.1	1329	/	0	38.89	565.2	0.76	1.24	0.97	0.59	9.38	28.8	0.37
	24	8.37	57.98	3056	11.8	0	47.83	930.3	1.74	1.46	1.28	0.79	22.2	33.1	0.57
	48	8.49	47.98	3666	10.8	0.002	54.83	1246	1.91	2.61	1.73	0.87	26.6	34.8	0.96
3	2	–	121.7	2885	26.3	/	197.1	35450	1.22	1.88	2.74	0.84	2.1	31	0.83
	6	8.24	134.2	2896	26	0.021	193.9	35750	5.53	2.75	2.7	0.89	2.43	28.6	0.98
	24	8.21	150.2	3000	12.2	0.009	198.1	35310	2	2.05	2.58	0.64	3.92	24	0.63
	48	8.27	152.2	3162	12.2	0.001	205.3	35320	1.67	1.51	2.61	1.04	4.33	22.3	0.85
4	2	8.38	70.71	4291	29.7	0.001	177	34840	1.99	2.75	2.41	0.72	3.27	25.8	1.77
	6	8.41	62.15	4395	36.4	0.008	186.8	39360	3.4	3.74	2.49	0.82	4.23	38.3	1.28
	24	8.37	23.71	5065	26.4	0.005	196.6	39250	3.66	3.6	2.37	2.03	4.61	23.8	1.18
	48	8.54	18.92	5248	16.1	0.001	200	38890	3.4	3.83	2.26	2.05	3.88	22.4	0.67
5	2	–	49.02	3507	26	0.005	187.6	36680	2.02	3.19	2.39	1.05	2.11	33.2	0.95
	6	8.3	42.2	3440	27.4	0.002	187.1	36590	1.96	3.04	2.55	0.91	1.9	23.9	2.23
	24	8.34	53.82	3475	25.6	0.011	191	37510	3.95	3.47	2.48	1.02	2.26	31.5	1.1
	48	8.36	49.83	3656	17.6	0	194.8	37330	2.98	3	2.52	1.97	2.15	23.8	0.85
6	2	6.18	21.52	669.9	99	0.035	28.19	995.8	1.27	1.53	0.64	1.33	350	59.7	1.67
	6	6.5	27.61	730.7	88	0.016	26.79	1037	1.28	1.1	0.95	1.34	360	34.2	2.47
	24	7.56	60.01	1116	92.9	0.002	32.71	1056	1.44	0.78	0.88	1.88	349	28.6	2.78
	48	8.44	128.1	1226	103	0.006	37.6	1109	1.47	1.14	0.99	2.03	332	31	1.18
	72	8.55	163.3	1279	84.5	0.003	41.54	1131	1.47	0.85	0.97	2.21	299	29.5	1.31
7	2days	8.49	323	838.5	/	0	37.16	460.8	0.69	0.71	0.32	0.99	11.6	40.5	0.83
	4days	8.39	259.9	907.8	/	0.004	42.84	493.2	0.64	0.53	0.22	1.13	12.8	16.9	0.4
	6days	8.33	207.7	972.1	/	0.001	44.19	512.6	0.84	0.64	0.01	1.89	15.1	20.7	1.11
8	4	7.93	63.61	2624	26.6	0.007	113.9	13910	2.14	2.14	1.68	1.97	5.21	25.3	0.59
	6	7.93	68.53	2701	25.5	0.009	131.4	14490	1.84	2.01	1.72	1.93	5.47	25.3	0.6
	24	8.09	91.82	3335	26.4	0.003	152.7	14760	1.67	1.73	1.92	2.42	6.42	23.9	1.61
	48	8.44	97.25	3429	15.5	0.003	174.4	15200	1.66	0.91	2	1.92	4.7	16.4	1.31
	72	8.46	89.39	3417	21.4	0.01	165.6	15100	3.6	2.01	2.49	0.84	2.93	19.6	1.06
	96	8.55	85.96	3505	17.7	0.007	176.3	15170	2.53	2.86	2.48	0.78	1.55	22.6	0.64
	120	8.48	84.22	3634	21.8	0.006	186.6	15530	2.42	2.31	2.45	0.79	1.41	22.4	0.57
9	2	6.15	14.65	583.5	91	0.054	12.28	826.2	5.96	9.07	0.26	1.48	356	191	4.36
	6	6.24	11.61	779	83.4	0.045	11.47	875.6	5.16	8.47	0.22	2.31	363	128	1.57
	24	6.34	7.624	767.2	65.8	0.036	12.74	856	4.41	7.53	0.24	2.16	353	90.4	1.54
	48	6.58	8.81	994.3	53.5	0.025	15.4	916	4.94	9.46	0.72	1.24	369	44	1.34
	72	6.97	11.37	969.9	42.8	0.017	16.9	915.8	4.74	9.4	0.71	1.08	366	33	1.47
10	2	6.05	24.7	573.6	86.5	0.035	9.26	838.8	4.98	13.5	0.06	1.79	331	236	1.92
	6	6.25	13.94	631.9	76.4	0.042	9.7	817.9	4.44	11.1	0.14	2.01	326	191	1.46
	24	6.42	10.74	1021	55.1	0.034	14.7	943.4	5.63	11.4	0.04	2.92	348	116	1.49
	48	6.58	12.81	1196	42.4	0.032	17.36	972.7	6.1	12.5	0.08	3.12	350	63.6	1.33
	72	6.73	16.24	1411	41.2	0.023	19.91	1029	6.92	16.2	/	3.67	360	46.6	1.37
11	2	11.16	1437	1367	60.1	0.014	15.98	72.54	1.92	1.87	2.25	−0.2	1.34	56.4	1.21
	6	11.03	1546	1651	63.3	0.006	22.36	47.76	1.86	1.42	2.58	0.04	0.45	40.1	1.52
	24	10.72	1663	1961	53.8	0.009	33.23	76.49	1.91	2.44	3.27	0.37	0.75	47.5	0.18
	48	10.12	1598	1936	53	0.006	39.53	73.8	1.92	2.65	3.42	0.5	0.61	37.6	0.22
12	2	5.96	21.76	888.8	224	0.062	14.63	1121	1.86	0.43	1.15	0.58	399	202	1.7
	6	6.13	12.15	918.3	169	0.039	19.49	1223	1.78	/	1.27	0.44	414	97.1	2.02
	24	6.36	13	1147	153	0.018	28.84	1424	1.75	0.17	1.4	0.48	425	46.3	1.67
	48	6.59	19.27	1384	141	0.021	34.18	1726	1.8	0.12	1.49	0.76	433	44.5	2.52
13	2	9.44	708.6	859.1	45.2	0.001	15.02	256.5	1.04	1.64	1.1	/	3.25	42.3	0.55
	6	9.2	709.9	847.7	37	0.001	19.37	273.5	1	/	1.17	/	5.17	35.9	1.64
	24	8.81	512	960.1	41.8	/	28.5	388.8	1.07	/	1.05	/	11.4	36.1	0.26
	48	8.43	361.1	1018	30.6	0.001	33.81	431.8	1.06	/	0.83	0.03	14.7	35.8	0.88
14	2	11.19	1317	1146	53.1	0.001	15.76	36.17	1.05	0.6	1.96	/	0.28	42.4	1.27
	6	11.15	1369	1244	49.7	/	19.06	70.39	1.11	0.64	2.12	0.12	0.31	38.1	0.45
	24	10.9	1531	1281	45.6	/	29.36	72.09	1.1	1.06	2.75	0.14	0.27	36.2	0.72
	48	10.97	1670	1421	45.6	/	43.02	60.52	1.15	1.42	3.19	0.22	0.33	36.8	0.6
15	2	9.19	481.2	827.7	44.7	0.006	15.31	283.7	1.1	2.19	0.97	/	6.26	29.9	0.24
	6	8.95	572.8	780.6	47.4	0.004	18.15	376.5	1	0.38	1.02	/	8.33	32.5	0.35
	24	8.57	461.5	871.5	46	0.003	26.75	639.7	1.08	/	0.95	/	11.9	33.7	1.67
	48	8.45	293.5	966	43.1	0.003	33.33	1144	1.11	0.39	0.85	/	18.2	34	0.56
Experimental set	Sampling time(h)	Eh (mV)	Ni	Cu	Zn	As	Se	Sr	Mo	Ag	Cd	Sb	Ba	Hg	Pb
1	2	209	4.3	2.791	21.28	4.243	3.505	925	4.11	0.05	0.08	7.46	189	0.34	1.98
	6	–	3.937	2.622	16.98	4.208	3.552	923	4.24	0.01	0.03	12.5	195	0.24	0.55
	24	183	3.421	2.606	11.73	3.677	3.491	876	4.45	0.01	0.03	14.6	180	0.27	0.36
	48	197	2.636	2.661	9.327	3.666	4.405	831	4.6	0	0.03	35.5	173	0.23	0.25
2	2	172	2.5	1.699	19.52	0.239	4.361	141	0.93	0.02	0.24	4.37	65	0.89	3.75
	6	–	1.74	1.095	24.25	0.356	5.304	170	0.95	0.01	3.98	6.7	82.9	0.19	4.91
	24	189	1.339	0.97	9.695	0.619	6.839	202	1.31	5.15	0.78	8.87	149	0.47	0.74
	48	173	1.089	0.935	9.672	0.731	8.08	214	1.56	0.22	0.5	16.3	159	0.19	0.76
3	2	190	4.023	2.223	17.63	3.981	2.376	890	3.87	0.08	0.05	6.47	180	0.14	1.03
	6	–	3.589	2.169	30.56	3.807	2.495	908	4.06	0.23	0.05	8.96	197	2.25	0.39
	24	198	2.858	2.067	24.38	3.472	2.331	900	4.13	0.06	0.03	5.43	204	0.92	0.28
	48	217	2.555	2.215	7.866	3.133	3.103	909	4.18	0.04	0.02	10.5	209	0.6	0.1
4	2	212	3.532	3.374	403.3	4.198	2.632	856	3.56	0.01	0.25	14.3	117	0.16	2.47
	6	194	3.686	3.404	252.4	4.546	2.914	975	4.1	0.03	0.14	9.6	137	0.93	1.39
	24	165	3.29	2.933	84.23	4.413	2.847	971	4.29	0.03	0.06	14	142	0.5	0.27
	48	171	3.417	3.008	19.48	4.31	2.62	953	4.27	0.02	0.04	5.65	147	0.39	0.21
5	2	220	5.304	2.338	29.45	4.57	2.498	911	4.03	0.02	0.08	6.09	158	0.44	1.92
	6	–	5.466	2.345	24.75	4.527	1.759	904	4.07	0.01	0.06	31.5	159	0.39	1.2
	24	247	5.679	2.572	13.21	4.237	2.582	941	4.23	0.1	0.04	12.3	186	1.33	0.85
	48	217	4.456	2.508	11.82	4.333	2.74	936	4.23	0.04	0.04	8.29	191	0.69	0.6
6	2	191	6.492	2.667	72.7	0.378	1.547	438	0.43	0.07	0.15	4.99	269	0.61	0.96
	6	261	6.33	2.141	30.69	0.374	1.542	457	0.49	0.02	0.1	22.8	281	0.44	0.46
	24	194	5.248	1.944	10.28	0.302	1.682	480	0.66	0.02	0.07	37.7	308	0.35	0.13
	48	161	4.902	2.05	16.32	0.302	1.432	510	0.81	0	0.1	5.46	339	0.3	0.17
	72	169	4.864	1.892	19.87	0.445	2.227	523	1.05	0	0.07	8.91	355	0.26	0.14
7	2days	160	5.444	2.521	64.97	0.227	3.489	200	1.18	6.77	1.01	10.1	158	0.53	4.44
	4days	166	1.027	1.033	14.42	0.116	3.585	244	1.47	0.19	0.08	6.35	213	0.23	0.65
	6days	160	1.001	0.956	26.14	0.195	2.436	245	1.53	0.06	0.07	21.4	224	0.19	0.21
8	4	157	2.937	3.201	39.59	1.864	1.814	430	5.14	/	0.08	7.95	180	0.11	0.46
	6	152	3.068	3.258	40.64	1.875	1.906	439	5.18	/	0.09	7.6	182	0.11	0.5
	24	158	2.533	2.179	85.69	1.871	1.985	469	5.35	/	0.07	29.9	191	0.06	0.37
	48	212	1.959	1.921	89.43	1.646	1.908	490	5.66	/	0.04	25.9	194	0.03	0.13
	72	200	1.778	2.202	90.86	1.734	2.252	492	5.84	0.17	0.04	21.4	193	0.49	0.06
	96	196	1.576	2.135	32.54	1.597	2.281	496	5.91	0.05	0.03	11.2	191	0.32	0.03
	120	124	1.536	2.251	32.65	1.637	2.504	503	6.15	0.04	0.03	9.56	194	0.16	0.07
9	2	141	5.433	2.504	38.24	0.95	2.186	443	0.66	0.01	0.07	80.6	262	0.01	0.29
	6	139	6.25	2.409	181.1	0.841	1.52	457	0.71	/	0.11	12.6	262	/	0.19
	24	137	5.498	2.04	35.26	0.732	1.985	445	0.74	/	0.06	11.5	257	/	0.02
	48	159	5.654	2.111	149.2	0.662	0.822	472	0.87	/	0.07	4.32	269	0.05	/
	72	246	5.588	2.075	56.71	0.591	1.247	481	0.98	/	0.07	8.5	270	/	/
10	2	155	5.935	2.814	137.5	0.832	1.226	423	0.59	/	1.81	18.7	256	/	0.55
	6	157	5.543	2.211	99.81	0.855	1.668	417	0.64	/	1.66	9.45	253	/	0.44
	24	137	5.812	4.67	215.3	1.059	2.012	445	0.76	/	1.6	11.8	264	/	0.08
	48	140	5.739	2.31	92.57	0.856	1.872	462	0.86	/	1.59	6.83	271	/	/
	72	192	5.785	2.483	92.67	1.031	1.993	479	0.89	/	1.6	5.66	283	/	/
11	2	184	0.409	0.957	34.47	0.535	2.847	42.9	1.22	0.11	0.06	25.5	15.5	0.52	0.78
	6	−2	0.22	0.642	1.745	1.089	5.1	66.9	1.43	/	0.01	30.8	26.4	0.28	0.05
	24	11	0.316	1.488	5.462	2.219	7.213	72.8	1.77	0	0	4.63	28.2	1.06	/
	48	48	0.15	1.634	12.51	2.568	8.484	68.3	1.98	0.01	0.01	6.15	25.9	1.02	/
12	2	142	7.963	2.471	150.5	0.449	1.419	453	0.51	/	0.22	6.26	273	0.12	0.46
	6	137	8.254	1.992	179.9	0.41	1.036	479	0.62	0	0.18	12.6	281	0.12	0.05
	24	195	8.59	1.871	101.2	0.227	0.988	507	0.81	0	0.26	4.46	297	0.11	0.03
	48	220	8.68	1.9	212.4	0.392	1.653	526	0.96	0.01	0.29	19.1	313	0.1	/
13	2	65	1.008	0.567	11.2	0.061	1.656	124	0.85	0.01	0.04	9.21	57.8	0.08	0.22
	6	70	0.589	0.352	1.018	0.007	1.68	134	0.95	0.02	0.02	30.5	66.7	0.06	0.01
	24	84	0.626	0.605	7.635	0.049	2.543	183	1.25	0.01	0.02	4.3	95.7	0.05	/
	48	100	0.741	0.521	8.015	0.154	3.211	203	1.43	0.02	0.02	14.8	108	0.05	/
14	2	−1	0.273	0.477	2.208	0.281	2.607	58.7	1.03	0.02	0.06	23.4	23.6	0.04	0.75
	6	−11	0.217	0.402	10.13	0.182	3.075	89.9	1.22	0.01	0.02	8.29	39.7	0.04	0.13
	24	8	0.158	0.817	11.09	0.664	5.605	89.9	1.51	0.01	0.01	14.7	40.2	0.06	0.04
	48	−5	0.223	1.354	1.835	0.648	6.411	93.2	1.71	0	0.01	12.8	39.5	0.3	/
15	2	103	4.982	0.812	9.383	/	1.574	129	0.89	0	0.04	4.85	61	0.58	0.2
	6	100	1.615	0.35	37.11	0.032	2.277	149	1	0	0.01	6.47	72	0.07	0.01
	24	99	1.422	0.451	10.69	/	1.286	192	1.14	0.01	0.03	30.6	96.7	0.08	/
	48	111	2.524	0.766	55.63	0.024	2.171	208	1.4	0.01	0.14	8.49	129	0.19	/

“/” – under detection limit; “-” - missing value.

**Table 6:** Trace element concentrations in leachate of coal host rock samples (μg/L).

Experimental set	Sampling time(h)	pH	Al	Si	P	Be	B	Ag	Sc	Ti	V	Cr	Mn	Fe	Co
1	2	–	191.9	3776	23.2	0.018	202.4	32170	1.96	4.42	12.5	0.88	3.38	44.8	3.31
	6	8.38	127.4	3999	22.6	0.009	216.2	31930	2.09	3.46	14.6	0.87	3.29	35.7	2.43
	24	8.46	115.8	4474	18.4	0.018	232.2	31390	2.41	3.67	18.8	0.94	1.81	37	2.24
	48	8.51	82.27	5284	8.96	0	242.2	31080	2.68	3.07	22.4	1.47	1.16	28	2.32
2	2	8.14	92.29	4822	27.5	0.005	207.4	34020	2.43	3.5	4.75	0.85	3.09	30	1.34
	6	8.14	77.68	5531	19	0.01	222.3	34150	2.8	3.86	6.15	0.82	3.58	34.4	0.97
	24	8.48	40.49	6693	1.59	0.003	235.9	32590	3.29	3.3	8.76	1.91	1.91	18	0.74
	48	8.55	44.31	7167	1.21	0.003	247.7	31530	3.48	3.16	11	1.82	0.62	19.3	0.55
3	2	–	99.45	3253	2.96	0.011	198.1	29960	1.61	2.69	11.7	0.74	3.48	26.4	2.95
	6	8.16	66.39	3598	/	0.009	205.3	30030	1.6	2.67	13.9	0.63	3.32	15.8	1.79
	24	8.14	66.64	4345	/	0.007	218.3	29820	1.92	5.06	17.2	0.63	3.37	23.2	2.57
	48	8.12	60.04	5160	/	0.007	225	29040	2.56	2.62	20.5	1.38	3.56	14.4	2.19
4	2days	7.74	10130	12940	10.6	0.351	67.31	717.3	10.6	409	32	10.2	8.63	795	3.04
	4days	7.72	6355	7515	/	0.227	69.1	1194	8.36	258	25.3	6.83	17.6	494	3.1
	6days	7.69	1207	5475	20.2	0.075	136.5	1407	3.38	47.4	15.1	0.63	22.4	86.5	1.91
5	2	2.09	808	2140	47.4	2.645	33.94	2713	1.05	3.38	13.1	1.59	93	514	29.9
	6	2.19	1363	3767	41.9	5.163	81.66	4484	1.89	2.41	21.8	1.74	189	1041	56.3
	24	2.3	3416	8226	48.1	9.791	145	7455	5.3	2.55	36	3.28	321	2421	123
	48	2.38	5030	10880	42.6	11.98	158.2	8321	9.16	4.53	43.4	4.52	365	3314	164
	72	2.42	6347	12170	45.1	13.69	171	8795	11.2	4.27	48.2	5.65	388	4099	190
6	2	6.93	1892	2599	17.3	0.093	13.79	100.5	1.91	86.5	6	2.37	1.43	104	2.13
	6	7.72	146	1252	16.8	0.073	27.5	339.7	0.34	1.88	4.02	0.98	5.36	35.4	2.97
	24	7.7	427.4	2514	14.4	0.121	67.04	239.8	1.51	8.26	10.6	/	3.29	49.2	3.87
	48	7.65	743	3799	16.5	0.334	83.54	549.1	2.43	24.2	14	0.11	15	103	3.91
	72	7.97	262.3	3879	/	0.021	97.52	747.8	2.26	12.6	13.8	/	10.3	/	0.37
7	2	11.24	3651	3649	36.1	0.439	9.808	241.4	3.05	37.1	23	1.86	3	209	4.9
	6	11.09	3987	3887	41.2	0.704	27.88	425.1	4.47	14.8	32	3.37	5.77	372	8.41
	24	10.61	4018	4417	33.1	0.868	74.6	432.3	4.65	18.6	53.4	2.15	5.71	361	9.03
	48	10.09	3505	4420	30.6	0.709	92.14	408.4	3.98	21.9	67.7	2.03	5.23	329	8.12
	72	9.51	3877	4880	31.2	1.029	103.5	561.1	5.35	25.2	76.5	2.8	9.63	522	12.1
	96	9.03	3029	4693	32.4	0.962	115	544	4.91	25.1	75	2.28	9.2	453	11
8	4	8.11	149.5	4360	16.3	0.019	138.5	12970	2.85	10.1	6.19	2.44	3.39	27.7	2.12
	6	7.84	65.81	4558	21.1	0.021	146.6	12840	3.5	5.17	7.74	2.57	4.88	52.7	2.08
	24	7.93	44.14	5450	16.9	0.009	176.8	12700	4.02	4.42	11.5	2.33	7.1	18.8	2.3
	48	8.01	39.93	5874	15.9	0.007	203.2	13050	4.01	4.04	15.2	1.67	5.37	20.8	1.59
	72	8.35	41.73	6236	13	0.009	205.4	12750	4.39	5.33	18	0.9	4.56	18	1.8
	96	8.46	51.58	6155	13.7	0.014	215.8	12930	4.59	7.13	20.1	0.52	3.77	22.7	1.33
	120	8.49	38.57	6411	14.4	0.005	221	12940	4.88	6.35	21.4	0.71	3.55	19.9	1.14
9	2	2.09	1419	1691	20.9	2.258	2.784	1678	4.02	11.2	3.75	1.65	18	207	1.82
	6	2.17	2682	3108	7.61	4.142	30.64	2900	3.93	9.82	6.09	1.86	31.2	376	2.78
	24	2.32	5781	7181	8.97	8.33	90.86	5065	6.59	8.98	8.32	3.09	58.2	863	5.98
	48	2.42	8313	10380	14.1	11.54	115.4	5871	9.06	11.2	7.16	4.13	74.4	1355	9.17
	72	2.47	10300	12740	10.4	13.17	123.5	6181	11.3	10.9	6.07	4.98	80.2	1735	11.9
10	2	2.07	787.9	2009	30.7	2.439	3.53	2580	3.55	9.96	12.4	1.58	104	514	30.8
	6	2.16	1341	3545	43.6	4.383	31.58	4555	7.83	11.3	21.4	2.14	212	990	58.3
	24	2.74	2893	6456	50.4	7.534	63.72	6169	9.27	11.7	34.4	3.34	330	2161	113
	48	2.3	4026	8787	48.6	9.596	79.4	6960	11.7	11.2	43.9	2.98	387	2871	145
	72	2.35	5198	10440	55	10.77	88.92	7487	15.1	12.8	51.2	3.9	417	3609	168
11	2	8.34	449.6	1456	29.6	0.109	24.2	79.03	1.51	7.54	3.5	/	0.62	50.7	0.52
	6	8.22	224.7	1788	35.5	0.052	34.6	309.9	1.66	2.29	3.76	/	1.21	38.3	0.4
	24	8.24	571	3429	31.9	0.032	54.17	392.6	2.61	29.6	6.12	0.07	1.33	53.7	0.46
	48	8.14	199.3	3769	37.4	0.013	66.09	537.1	2.79	9.72	7.19	/	1.23	37.2	1.29
12	2	11.05	2688	2950	53.7	0.317	20.45	215.9	5.34	11.5	23.6	0.65	2.27	128	4.06
	6	10.88	3031	3727	54.8	0.369	33.68	387.4	4.5	17.3	35.6	1.01	2.93	174	4.75
	24	10.5	2631	4009	61.1	0.308	55.41	399.9	4.33	9.13	54.6	1	2.59	174	4.13
	48	10.37	2333	4124	59.1	0.353	68.86	468.9	4.38	11.6	69.1	1.2	3.81	208	5.25
13	2	5.45	626	1426	47.1	0.168	13.73	309.7	1.4	24.9	2.61	0.12	4.27	66.8	2.46
	6	6.27	234.3	1321	42	0.149	25.17	383.3	1.23	6.73	2.69	/	5.68	49.4	3.04
	24	6.79	75.01	2181	49.1	0.079	45.92	765.4	1.72	2.47	4.14	/	9.92	36.5	3.38
	48	6.95	49.3	3102	42	0.026	59.92	1418	2.26	2.86	5.27	/	20.1	34.8	2.49
Experimental set	Sampling time(h)	Eh (mV)	Ni	Cu	Zn	As	Se	Sr	Mo	Ag	Cd	Sb	Ba	Hg	Pb
1	2	169	5.238	3.378	18.94	6.386	5.874	1296	5.89	0.08	0.09	33.9	28.5	0.18	2.21
	6	–	4.352	2.917	19.23	7.571	6.397	1305	6.71	0.03	0.05	18.5	28.2	0.18	0.68
	24	179	3.935	3.104	9.664	9.687	8.359	1299	8.03	0.06	0.04	20	30.7	0.13	0.48
	48	211	2.967	3.11	8.594	11.49	10.51	1271	8.41	0.04	0.03	27.8	30.3	0.14	0.28
2	2	193	2.686	5.734	225.9	3.579	2.435	1059	3.79	0.02	0.13	10.9	22.4	0.15	1.57
	6	189	2.414	2.317	137.7	3.305	3.735	1104	4.02	0.02	0.1	6.71	21.5	0.12	0.94
	24	156	1.887	2.033	12.14	3.335	7.075	1125	4.58	0.02	0.02	9.81	19.7	0.13	0.39
	48	161	1.609	2.235	4.986	3.563	9.482	1111	5.15	0.01	0.03	6.6	20	0.11	0.11
3	2	182	4.62	2.868	29.54	6.213	5.089	1211	5.77	0.04	0.07	28.6	25.4	0.44	1.81
	6	–	3.566	2.505	15.8	6.986	5.732	1226	6.45	0.02	0.04	6.6	24.3	0.31	0.58
	24	193	3.284	2.607	6.424	8.814	7.86	1234	7.63	0.01	0.03	22.1	25.3	0.26	0.41
	48	214	3.027	2.631	7.766	10.05	8.782	1278	8.39	0.01	0.03	17.5	25	0.25	0.36
4	2days	160–181	8.827	4.861	25.46	6.209	6.152	58.9	4.65	0.29	0.09	7.64	13.3	0.21	4.21
	4days	188	5.521	3.712	30.2	9.766	8.097	123	5.34	0.14	0.06	16	9.7	0.18	2.24
	6days	196–208	2.763	1.355	11.07	8.578	9.233	155	5.7	0.04	0.06	9.39	3.47	0.45	0.84
5	2	399	29.25	26.52	264.8	3.119	1.049	341	0.13	0.07	0.37	40.6	7.79	0.19	14.9
	6	396	59.54	53.34	41.12	5.509	1.731	600	0.11	0.15	0.56	13.9	15	0.17	29.1
	24	382	140.1	133.6	93.2	8.27	2.644	977	0.13	0.2	1.02	15	27.5	0.21	71.6
	48	391	192.2	207.6	139.5	9.908	3.055	1101	0.17	0.29	1.38	5.98	35.3	0.23	102
	72	402	230.5	276.1	214.9	10.42	3.895	1153	0.22	0.43	1.65	4.97	40	0.19	126
6	2	188	1.107	0.935	32.08	1.03	2.069	7.16	1.64	0.09	0.03	34.6	2.38	0.03	0.84
	6	174	1.2	0.887	29.27	1.267	2.569	43.6	2.41	/	0.04	34.5	0.65	/	0.91
	24	149	1.552	2.22	9.857	3.836	4.675	29.7	3.77	/	0.04	47.2	1.12	0.04	2.27
	48	232	4.546	2.929	24.14	6.844	6.287	71.6	4.33	/	0.06	4.38	1.93	0.03	1.07
	72	207	0.422	0.309	0.881	8.897	7.533	91.4	5.4	/	0.01	5.68	0.83	0.01	/
7	2	−2	6.332	7.414	14.02	7.394	3.83	23.2	2.34	0.01	0.08	9.19	3.93	/	9.1
	6	11	10.42	14.44	64.45	12.04	6.099	45.6	2.93	0.04	0.12	11.9	5.22	/	13.7
	24	177	12.36	13.22	14.37	24.38	10.2	47.5	4.37	/	0.1	9.74	5.1	1.03	16
	48	96	9.82	12.09	14.63	32.11	12.13	49.4	4.93	0.07	0.09	12.4	4.79	1.04	14
	72	110	15.2	18.35	14.34	38.02	14.41	67.8	4.87	0.06	0.1	11.2	6.67	/	20.3
	96	93	12.52	17.12	17.74	39.3	14.64	73.3	5.56	0.24	0.1	12	5.98	/	18.3
8	4	172	3.223	3.203	28.48	4.581	4.556	508	6.72	0.11	0.07	17	28.8	0.19	0.75
	6	163	3.056	3.383	86.67	5.497	5.275	587	7.44	0.03	0.09	13.4	25.3	0.13	0.86
	24	164	3.149	3.156	112.5	8.152	6.094	675	8.76	0.01	0.12	12.8	24.3	0.06	0.3
	48	222	2.461	2.819	35.61	8.804	7.135	733	9.55	0	0.07	9.39	22.1	0.03	0.1
	72	232	2.201	2.694	20.81	9.17	8.724	758	9.62	/	0.05	19.2	21	0.03	0.05
	96	211	2.189	2.707	23.16	9.636	8.336	798	10.2	/	0.06	10.9	21.2	0.03	0.15
	120	116	2.162	2.837	20.97	9.724	9.815	826	10.4	/	0.07	7.81	20.9	/	0.08
9	2	389	13.56	7.19	320.9	0.643	1.732	230	0.31	0.01	0.26	8.5	11.8	0.17	5.47
	6	452	7.749	10.73	33.03	0.336	0.981	391	0.27	0.07	0.16	13.5	23	/	6.41
	24	398	12.56	42.58	44.1	0.164	0.625	685	0.27	0.19	0.15	6.99	49.7	/	14.6
	48	398	19.11	101.7	53.68	0.318	1.078	825	0.29	0.35	0.26	8.94	69.6	/	25.2
	72	400	22.56	156.8	81.68	0.381	0.966	883	0.29	0.21	0.26	20.7	85.4	/	36.2
10	2	398	32.4	29.59	80.96	4.754	2.019	343	0.37	0.19	0.42	23.5	9.19	/	17.6
	6	391	64.64	53.22	430.3	7.853	3.848	608	0.56	0.33	0.7	13.2	16.2	0.61	27.3
	24	425	133.4	116	204.3	11.07	4.166	867	0.38	0.08	1.06	20.1	24.5	0.24	55.6
	48	411	179.3	173	245.9	13.22	4.627	981	0.4	0.05	1.32	11.1	30.6	0.1	77.9
	72	415	209.6	217.2	329.8	14.78	4.41	1031	0.41	0.11	1.47	5.94	35.1	0.07	96.1
11	2	110	0.338	0.985	16.17	/	0.909	7.06	0.28	0.02	0.05	10.3	0.98	0.04	1.08
	6	116	0.226	0.427	1.436	/	0.795	34.7	0.41	0.02	0.03	7.55	0.9	0.04	0.35
	24	110	0.26	0.342	2.825	/	2.818	47.1	0.77	0.01	0.03	9.91	1.27	0.07	0.16
	48	114	0.138	0.124	5.579	0.169	5.336	65.4	1.13	0.01	0.01	24.3	0.89	0.05	/
12	2	11	4.608	4.997	14.38	6.597	4.079	20.3	2.37	0.46	0.31	14.2	2.67	0.53	8.84
	6	9	5.617	6.708	39.81	11.62	6.577	42.6	3.18	0.16	0.12	8.94	3.13	0.28	8.11
	24	35	4.743	5.051	20.66	20.66	10.21	43.4	4.4	0.09	0.09	16.1	2.36	0.21	5.82
	48	20	5.55	6.517	27.2	27.98	12.4	53.7	5.17	0.02	0.09	27.9	2.67	1.06	6.79
13	2	202	2.538	0.468	16.57	0.203	1.252	34.1	0.71	0.02	0.09	11.3	1.64	0.04	0.42
	6	175	2.784	0.433	25.38	0.5	2.733	43.1	1.63	0.01	0.08	12.9	1.04	0.04	0.16
	24	172	2.733	0.264	20.53	1.027	4.053	81.4	3.07	0.01	0.11	13.3	1.25	0.02	0.05
	48	158	2.587	0.295	28.84	2.116	5.012	148	3.75	0	0.1	5.7	1.6	0.09	/

“/” – under detection limit; “-” - missing value.

## 2 Experimental design, materials, and methods

### 2.1 Description of the sampling area

**Fig. 1:**
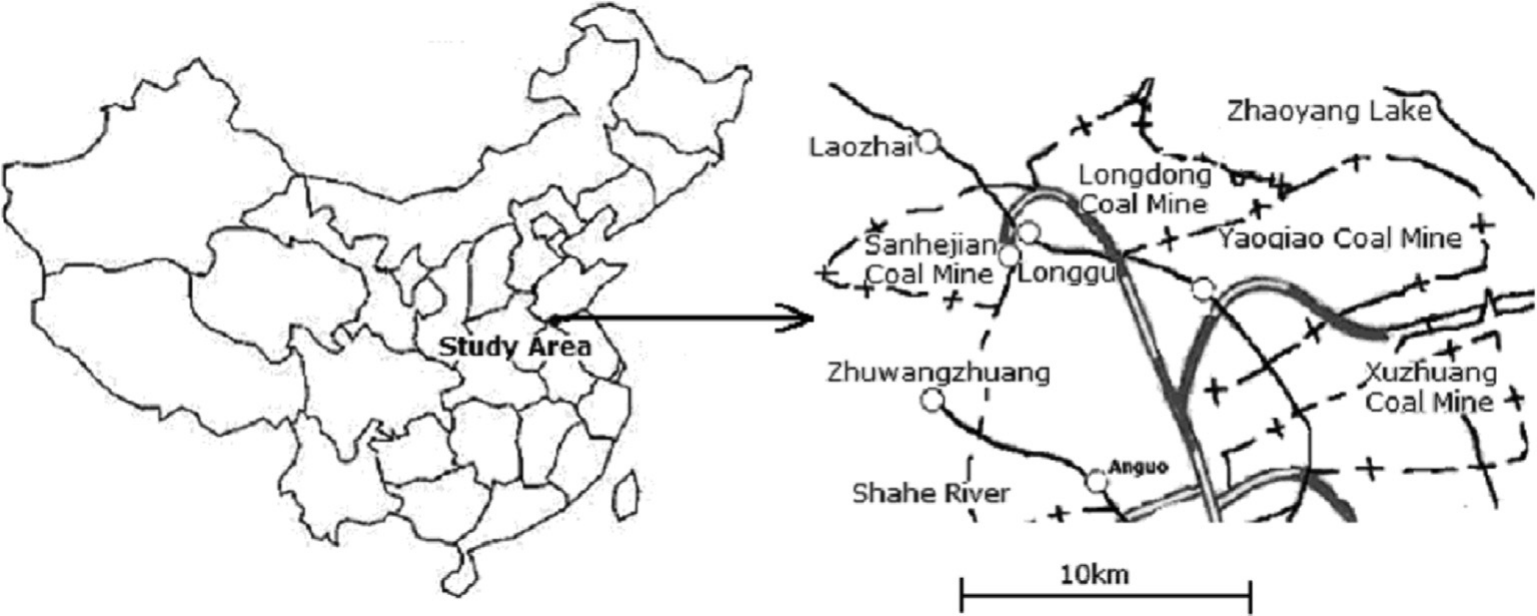
Location of the study area.

Solid and water samples were collected at Xuzhou-Datun coal mine district, which is located at the north of Jiangsu province, eastern China (Fig. 1). Average temperature, relative humidity and air pressure in the region are 14 °C, 73%, 101280 Pa, respectively. Average precipitation is 758 mm, ranging from 492 mm to 1178 mm, with an average evaporation 1623 mm. The geology in this area could be described as a series of sediment stratum cover the Archean system, from bottom to top these are Simian, Cambrian, middle-lower Ordovician, middle-upper Carboniferous, Permian, Jurassic, Cretaceous, Tertiary and Quaternary system.

The coal seams that are being mined are located in the Carboniferous and Permian systems, the former includes Benxi and Taiyuan formations, and the latter includes Shanxi and Lower-Shihezi formations, listed from bottom to top in both systems. The existing coal seams are located in the Shanxi formation (coal seam No. 7&9) and Lower-Shihezi (coal seam No. 2) Formation. In the lower formation, white feldspar, quartz granule-sandstone and silicon-mudstone cementation are the main minerals. Grey mudstone, sand-mudstone and sandstone are the main rocks in the middle Shanxi formation with some silicon-mudstone and siderite also present [2]. In the upper formation, grey-green middle-fine quartz sandstone, siltstone, mudstone are the main minerals. In this area, ground water was reported to be contaminated by coal mining and electricity plants [3], [4].

### 2.2 Sampling

To investigate contamination of trace elements on surface and ground water by coal mining activities, 28 solid samples and 16 ground water and surface water samples were collected. The solid samples were collected from roof, floor, and coal from the working areas of the coal seam No. 2, 7, and 9 as shown in Table 1, Table 2, Table 3, Table 4 The ground water samples, including that from roof leaching, limestone aquifer and caved goaf, were collected in coal mines. Surface water samples were collected from lakes that located in both coal mine and non-coal mine district. 1000 mL Nalgene bottles were used to contain water samples, which were cleaned by acid in laboratory and rinsed twice before the sample were collected. A JENCO 6010 pH/ORP meter was used to test Eh and Eh value of samples. Coal and host rock samples were collected from the working area and put into black plastic bags and sealed immediately.

### 2.3 Experiments

Some factors may control or impact the leaching process, leading to different migration behavior of major and trace elements. pH value is a key parameter in leaching behavior of trace elements from coal and host rock. Most of leaching study focus on the acid leaching behavior for the higher mobility of metal elements [5], [6], [7], [8], [9]. The group of Zhang have applied a series of experiments to investigate acid leaching of vanadium from coal [7], [8], [9]. However, metalloid elements may release more in an alkaline environment [6]. At the same time, high pH values are usually observed in coal mine water. Eh impact the leaching behavior in two aspects, higher kinetical ratio in one hand, and oxidation of some minerals that stable in an anaerobic environment in the other hand [5]. Temperature may promote the water-rock interaction according to the thermodynamics laws [10]. Some researchers have also discussed the impact of liquid/solid ratio [7], types of leaching water [5] on the trace elements’ migration, as some major components, such as sulfate, may hider release of trace elements [5].

The leaching experiments were designed in batch mode to simulate water-rock interaction in a coal seam where the water moves slow and reaction tends to achieve equilibrium [7], [11], [12], [13]. To avoid effect of content of solid samples on leaching behavior, most of the leaching experiments used the same coal/rock samples. All the glassware used in the experiments were soaked in 3.2 mol/L HNO_3_ for two days so as to reduce cross-contamination. The selected solid samples were ground until 75 μm. 30 mg were weighted for each leaching experiment, for interacting with 1000 mL aliquot water. The water used in the experiments were ultra-pure water, surface water from coal mine district and non-coal mine district to simulate different environment. To investigate the immigration behavior of trace elements in both acid and alkaline environment, initial pH of ultra-pure water was set to 2, 5.6, 7 and 12. Temperature was controlled using a water bath at 38 °C, or in raw temperature, which was about 15 °C. To simulate a ‘closed environment’ with low pO_2_, (see Stumm and Morgan (1996) for details) [14], bottles were closed with a rubber stopper. Flasks were sealed and shaken every two hours. Leachate solutions were collected using syringes at 2, 6, 24 and 48 hours as the reaction may achieve equilibrium in hours in some conditions [7], some samples were collected even later to ten days to observe long time behavior [6]. 0.5 mol/L HNO_3_ was added into each sample to reduce adsorption and hydration of trace elements. The pH and Eh of the solution during experiments was determined by a JENCO 6010 pH/ORP meter. The conditions in every experimental setting are illustrated in Table 7, Table 8 for coal leaching and host rock leaching, respectively.

**Table 7:** The experimental settings for the coal leaching.

Experimental set	Solid sample	Solid sample No. in the Table 2	Type of the leaching water	Initial pH	Open/closed	Temperature (°C)
1	Coal	17	Surface water (coal mine district)	8.68	Open	15.7
2	Coal	5	Ultra-pure water	8.85	Open	38
3	Coal	17	Surface water (coal mine district)	8.68	Closed	38
4	Coal	17	Surface water (coal mine district)	8.68	Open	16.2
5	Coal	17	Surface water (coal mine district)	8.68	Closed	17.5
6	Coal	17	Ultra-pure water	2	Open	38
7	Coal	5	Ultra-pure water	7	Open	38
8	Coal	17	Surface water (non-coal mine district)	8.80	Open	38
9	Coal	17	Ultra-pure water	2	Open	17.5
10	Coal	17	Ultra-pure water	2	Closed	16.5
11	Coal	17	Ultra-pure water	12	Open	38
12	Coal	17	Ultra-pure water	2	Closed	38
13	Coal	17	Ultra-pure water	5.6	Open	38
14	Coal	17	Ultra-pure water	12	Closed	38
15	Coal	17	Ultra-pure water	5.6	Closed	38

**Table 8:** The experimental settings for the host rock leaching.

Experimental set	Solid sample	Solid sample No. in the Table 4	Type of the leaching water	Initial pH	Open/closed	temperature
1	Roof	14	Surface water (coal mine district)	8.68	Open	38
2	Floor	4	Surface water (coal mine district)	8.68	Open	38
3	Roof	14	Surface water (coal mine district)	8.68	Closed	38
4	Roof	1	Ultra-pure water	7	Open	38
5	Roof	14	Ultra-pure water	2	Open	38
6	Roof	14	Ultra-pure water	5.6	Open	38
7	Roof	14	Ultra-pure water	12	Open	38
8	Roof	14	Surface water (non-coal mine district)	8.80	Open	38
9	Floor	4	Ultra-pure water	2	Open	38
10	Roof	14	Ultra-pure water	2	Closed	38
11	Floor	4	Ultra-pure water	5.6	Open	38
12	Roof	14	Ultra-pure water	12	Closed	38
13	Roof	14	Ultra-pure water	5.6	Closed	38

While the solid sample were digested, 0.05 mg of samples were carefully weighted and put into tetrafluoroethylene crucibles, 3 mL of HNO_3_, 1mL of HF and 1 mL of HClO_4_ were added and heated until all liquid consumed up. Then 3 mL of HCl were added and heated again until the consumption of the liquid. After that, the crucibles were rinsed using ultra-pure water, and then filter into a constant volume of 50 mL using volumetric flasks for analysis.

### 2.4 Testing

The data of ground water and surface water samples are shown in related article [1]. The data of solid samples and the water samples collected from the leaching experiments are shown in the DiB article, as supporting material for the related article. In the related article, selected attributes were used based on their concentration levels, proportion of under-detected data, and feature engineering.

Major ions and physical parameters of water samples were determined in Jiangsu Provincial Coal Geology Research Institute in line with Chinese standard protocols. pH was tested both in site and in the laboratory using Glass electrode method (GB/T 6920-86). Total dissolved solids and hardness were analyzed in term of the standard GB/T 8538 method. Mg and Ca were measured using atomic absorption spectrophotometric (GB 11905-89). K and Na were analyzed by flame atomic absorption spectrophotometry (GB 11904-89). Fe, Ammonia and Nitrate-Nitrogen were analyzed by phenanthroline spectrophotometry (HJ/T 345– 2007), phase molecular absorption spectrometry (HJ/T 195–2005) and gas-phase molecular absorption spectrometry (HJ/J 198–2005), respectively. Sulphate and chloride were determined using flame atomic absorption spectrophotometry (GB 13196-91) and silver nitrate titration (GB 11896-89), respectively.

Concentration of trace elements in solid and liquid samples were determined by ICP-AES and ICP-MS. The ICP-AES analysis was carried out in the Nanjing University using a JY38S ICP-AES model. Limit of detection and deviation for the analysis were 0.01 μg/mL and less than 2%, respectively. The ICP-MS analysis was carried out in the Analysis and Test Centre of China University of Mining and Technology using the X-Series ICP-MS-Thermo Electron Co., Rh was used as internal standard to determine limit of detection (0.5 pg/mL) and analytical deviation (less than 2%).
