# Supplementary material for: Data on trace element concentrations in coal and host rock and leaching product in different pH values and open/closed environments
Source: Data Brief. 2019 May 23;25:104053. doi: 10.1016/j.dib.2019.104053 (PMC6556551; doi:10.1016/j.dib.2019.104053)
Supplement: Multimedia component 1 [file mmc1.docx]

Dear Prof [Prosun Bhattacharya](https://www.journals.elsevier.com:443/groundwater-for-sustainable-development/editorial-board/prosun-bhattacharya), Editor-in-Chief:

We have checked that there is no conflict of interest exits in the submission of this manuscript, and manuscript is approved by all authors for publication. I would like to declare on behalf of my co-authors that the work described was original research that has not been published previously, and not under consideration for publication elsewhere, in whole or in part. All the authors listed have approved the manuscript that is enclosed.

Thank you and best regards.

Yours sincerely,
Yao SHAN

Corresponding author:
Name: Yao SHAN
E-mail: 9106350@qq.com
